# Potential of platinum-resensitization by Wnt signaling modulators as treatment approach for epithelial ovarian cancer

**DOI:** 10.1007/s00432-020-03317-4

**Published:** 2020-07-17

**Authors:** Till Kaltofen, Valentina Preinfalk, Stephanie Schwertler, Patricia Fraungruber, Helene Heidegger, Theresa Vilsmaier, Aurelia Vattai, Bastian Czogalla, Doris Mayr, Sven Mahner, Udo Jeschke, Fabian Trillsch

**Affiliations:** 1 Department of Obstetrics and Gynecology, University Hospital, LMU Munich, Munich, Germany; 2 Department of Pathology, University Hospital, LMU Munich, Munich, Germany; 3 Department of Obstetrics and Gynecology, University Hospital Augsburg, Augsburg, Germany

**Keywords:** Ovarian cancer, Platinum-resistance, Wnt, β-catenin, Snail/ slug, E-cadherin

## Abstract

**Purpose:**

Canonical Wnt/ β-catenin pathway is one mechanism being activated in platinum-resistant epithelial ovarian cancer (EOC). Detecting potential targets for Wnt pathway modulation as a putative future therapeutic approach was the aim of this study.

**Methods:**

Biological effects of different Wnt modulators (SB216763, XAV939 and triptolide) on the EOC cell lines A2780 and its platinum-resistant clone A2780cis were investigated via multiple functional tests. Immunohistochemistry (IHC) was carried out to compare the expression levels of Wnt marker proteins (β-catenin, snail/ slug, E-cadherin) in patient specimens and to correlate them with lifetime data.

**Results:**

We could show that activated Wnt signaling of the platinum-resistant EOC cell line A2780cis can be reversed by Wnt manipulators through SB216763 or XAV939. All Wnt manipulators tested consecutively decreased cell proliferation and cell viability. Apoptosis of A2780 and A2780cis was enhanced by triptolide in a dose-dependent manner, whereas cell migration was inhibited by SB216763 and triptolide. IHC analyses elucidated significantly different expression patterns for Wnt markers in the serous subtype. Herein, higher plasmatic snail/ slug expression is associated with improved progression-free (PFS) and overall survival (OS).

**Conclusion:**

According to the described effects on EOC biology, all three Wnt manipulators seem to have the potential to augment the impact of a platinum-based chemotherapy in EOC. This is promising as a dominance of this pathway was confirmed in serous histology.

**Electronic supplementary material:**

The online version of this article (10.1007/s00432-020-03317-4) contains supplementary material, which is available to authorized users.

## Introduction

Epithelial ovarian cancer (EOC) is the leading cause of death from gynecologic malignancies and the seventh most common cancer in women worldwide (International Agency for Research on Cancer 2020). With a relative five-year survival rate of 41% across all Federation of Gynecology and Obstetrics (FIGO) stages, survival is poor. Diagnosis is usually made in advanced FIGO stage III and IV in 75% of the patients (Jayson et al. 2014). In case of relapse, EOC will develop platinum-resistance over time and is driven by a range of heterogeneous primary and acquired mechanisms in different signaling cascades (Freimund et al. 2018; Rabik and Dolan 2007).

**Fig. 1:**
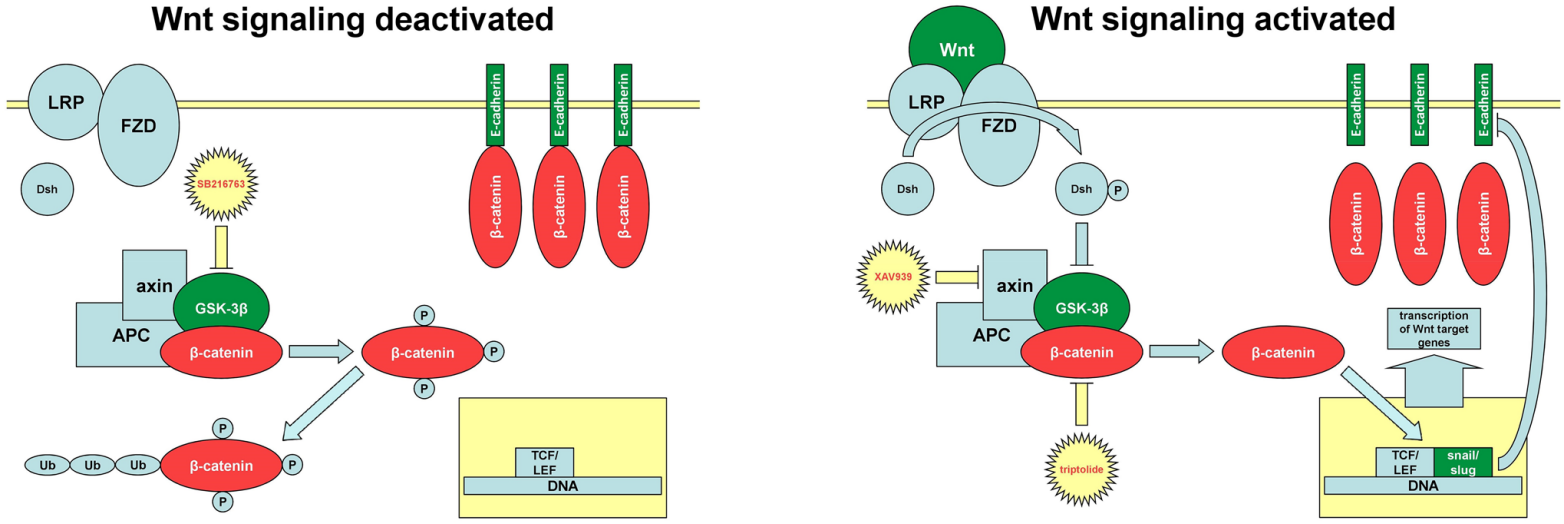
Canonical Wnt signaling pathway: In absence of Wnt ligand (left), the destruction complex, consisting of GSK-3β, adenomatous polyposis coli (*APC*) and axin, hyperphosphorylates β-catenin, which marks it for ubiquitination (*Ub*) and proteasomal degradation. Binding of Wnt ligand (right) to a frizzled (*FZD*)/lipoprotein receptor-related protein (*LRP*) receptor complex leads to the phosphorylation of dishevelled (Dsh) inactivating GSK-3β and preventing the phosphorylation of β-catenin. Non-phosphorylated β-catenin shifts into the nucleus and forms a complex with T-cell factor (*TCF*)/lymphoid enhancer factor (*LEF*) activating transcription of Wnt target genes. Furthermore, the expression of zinc-finger transcription factors snail and slug is upregulated. They bind to E-boxes in E-cadherins promoter region and prevent its transcription. Suppresion of E-cadherin offers more available cytoplasmic β-catenin and induces a self-driven positive feedback loop. In conclusion, by losing E-cadherin as an adhesion molecule cells undergo EMT (Arend et al. 2013; Gasior et al. 2017; Gatcliffe et al. 2008). The three inhibitors (SB216763, XAV939 triptolide) used in this study, are shown in their typical domain

One of them is considered to be the canonical Wnt/ β-catenin pathway (Fig. 1) (Barghout et al. 2015; Nagaraj et al. 2015), which is essential for the development and integrity of all multicellular organisms (Wiese et al. 2018) but dysregulation of the pathway has the potential to promote various diseases including EOC (Arend et al. 2013; Nusse and Clevers 2017; Shang et al. 2017). Except for endometrioid ovarian cancer (OC), mutations in canonical Wnt signaling in EOC are rarely seen (Kim et al. 2008), so that in contrast a general activation of the cascade has been considered to promote tumor progression (Dubeau 2008; Gatcliffe et al. 2008). However, conflicting results indicate a possible inhibitory role for tumor progression from time to time (Bodnar et al. 2014; Seagle et al. 2016).

It has been described that inhibition of canonical Wnt signaling can re-sensitize platinum-resistant EOC cells to platinum, which, therefore, represents a promising target for therapeutic approaches. The general knockdown of β-catenin, a key regulator in canonical Wnt signaling being located in the nucleus, reinduced chemosensitivity to platinum in cisplatin-resistant OC cells in vitro (Nagaraj et al. 2015). Additional, inhibitors of the Wnt signaling cascade exhibited potential to re-sensitize EOC cells to platinum: iCG-001/ PRI-724 (Nagaraj et al. 2015), CCT036477 (Barghout et al. 2015), WNT974 (Boone et al. 2016) or triptolide (Rivard et al. 2014; Westfall et al. 2008). However, their effects have not been independently proven and their possible clinical implications have not been systematically followed.

Within this study, the clinical and prognostic significance of Wnt signaling markers have been investigated to elucidate and confirm specific targets for Wnt pathway modulations with consecutive therapeutic perspectives. Besides the investigation of the protein expression of Wnt markers, the Wnt modulators SB216763, XAV939 and triptolide were applied to investigate their effects on the cell biology of the EOC cell line A2780 and its platinum-resistant clone A2780cis and to test the ability for putative therapeutic approaches by increasing sensitivity to platinum.

## Methods

### Experiments on cultured cells

#### Cell culture

The EOC cell line A2780 and its platinum-resistant clone A2780cis (European Collection of Authenticated Cell Cultures, Salisbury, UK) were cultured in Roswell Park Memorial Institute 1640 medium with GlutaMAX (Gibco, Gaithersburg, MD, USA) supplemented with 10% fetal bovine serum (Biochrom, Berlin, Germany) at 37 °C in the presence of 5% CO_2_ in cell culture flasks. A2780cis cells were treated with 1 µM carboplatin at each change of medium to maintain resistance. No antibiotics or antimycotics were used.

#### Wnt modulators

XAV939 is a small molecule inhibitor of Wnt signaling pathway. It acts through binding and inhibiting tankyrase's catalytic poly ADP ribose polymerase (PARP) domain, which usually destabilizes axin as part of the destruction complex (Chen et al. 2009; Wu et al. 2016) (Fig. 1). Wu et al. showed the resensitization to chemotherapy of colon cancer cells through XAV939 treatment (Wu et al. 2016). Furthermore, in human ovarian cancer cells, this tankyrase inhibitor was able to overcome chemoresistance driven through overexpression of long non-coding RNA (Li et al. 2016).

SB216763 is an inhibitor of the glycogen synthase kinase 3β (GSK-3β) and following this, in contrast to XAV939, an activator of the Wnt/ β-catenin pathway (Naujok et al. 2014) (Fig. 1). Indeed, inhibition of the GSK-3β was also shown to inhibit cancer cell proliferation (Cao et al. 2006; Schulz et al. 2018), maybe attributable to several recent studies describing the GSK-3β as a tumor promoter besides its typical role in Wnt pathway. So far, the serine-threonine kinase has multiple functions in different regulatory mechanisms or pathways and act as a chameleon in cancer's context (Domoto et al. 2016; Patel and Woodgett 2008).

We also included triptolide, a diterpenoid triexpoxide with multiple actions in eucaryotes, in our study. Its antineoplastic impact was already shown for different solid tumors, for example, breast, bladder, stomach or ovary (Shao et al. 2014; Yang et al. 2003). Specific molecular mechanisms are still under debate. One is the degradation of β-catenin (Fig. 1) as shown in breast cancer cells (Shao et al. 2014).

#### Cell proliferation and viability assay

5 × 10^3^ cells/well (A2780 and A2780cis) were seeded in 96-well plates overnight and afterwards incubated with different concentrations of SB216763 (12.5 µM, 25 µM, 50 µM, 100 µM) (Sigma-Aldrich, Taufkirchen, Germany), XAV939 (6.25 µM, 12.5 µM, 25 µM, 50 µM) (Sigma-Aldrich) and triptolide (6.25 nM, 12.5 nM, 25 nM, 50 nM) (Sigma-Aldrich).

S-phase-dependent synthesis of DNA during the cell cycle and, therefore, cellular proliferation was analyzed with thymidine analog 5-bromo-2′-deoxyuridine (BrdU) enzyme-linked immunosorbent assay (ELISA) after 72 h. Cell viability was determined using a 3-(4,5-dimethylthiazol-2-yl)-2,5-diphenyltetrazolium bromide (MTT) (Sigma-Aldrich) colorimetric assay after 48 h and 72 h. The procedures of both techniques were already reported by our colleagues (Geiger et al. 2016; Zhu et al. 2018).

Controls of untreated cells (for incubation with triptolide), cells treated with 2‰ (for incubation with SB216763) and 5‰ (for incubation with XAV939) dimethyl sulfoxide (DMSO) were carried out. For the evaluation the control without DMSO was set 100%.

#### M30 CytoDEATH apoptosis assay

The M30 CytoDeath apoptosis assay is used for determination of early apoptosis. A specific epitope of cytokeratin 18, which is presented after cleavage by caspases during apoptosis, is detected. OC cells were grown on microscope slides to subconfluency, incubated for 48 h in the presence of triptolide at different concentrations (6.25 nM, 12.5 nM, 25 nM, 50 nM and without as control), fixed and stored at − 20 °C. After thawing, washing and incubation with M30 CytoDEATH antibody (Alexis, San Diego, CA, USA) (Table 1) overnight immunocytochemical evaluation using ZytoChem-Plus HRP Polymer-Kit (Zytomed Systems, Berlin, Germany) and 3,3′-diaminobenzidine as chromogenic substrate (DAB) (Carl Roth, Karlsruhe, Germany) followed. Images were captured with a microscope including a digital camera system (Leica, Wetzlar, Germany).

**Table 1:** Antibodies used in this study: Stated are only antibodies, not part of a laboratory kit

Antigen	Antibody	Dilution	Detection system	Chromogenic substrate
β-catenin	Anti-β-catenin (rabbit IgG)	1:600	Vectastain Elite rabbit-IgG-Kit	AEC
		1:300	ZytoChem-Plus HRP Polymer-Kit	DAB
E-cadherin	Anti-E-cadherin (mouse IgG)	1:100	ZytoChem-Plus HRP Polymer-Kit	DAB
M30	Anti-M30 (mouse IgG)	1:50	ZytoChem-Plus HRP Polymer-Kit	DAB
Snail/ slug	Anti-snail/ slug (rabbit IgG)	1:800	ZytoChem-Plus HRP Polymer-Kit	DAB

#### Cell death detection ELISA

Cell death was quantified with the sandwich-enzyme-immunoassay-method of Cell Death Detection ELISA^PLUS^-Kit (Roche, Basel, Switzerland) according to the manufacturer's protocol. Following induced cell death, mouse monoclonal antibodies bind against cytoplasmic histone-associated DNA-fragments (mono- and oligonucleosomes). In brief, 5 × 10^4^ cells/well were grown overnight in 96-well culture plates and then incubated with 6.25 nM, 12.5 nM, 25 nM and 50 nM of triptolide for 24 h. Details on the further procedure can be found in Geiger et al. (Geiger et al. 2016). The apoptotic index is presented as an enrichment factor, which is calculated as absorbance of sample cells divided by absorbance of control cells without triptolide (enrichment factor = 1.0).

#### Wound healing assay

The assays were performed according to the protocol in Zhu et al. (2018). After the standardized scratching on A2780 and A2780cis monolayers, we added 100 µM SB216763, 50 µM XAV939 or 50 nM triptolide. Controls were carried out in parallel to cell proliferation and viability assay. Afterwards, cell migration was documented by measuring the wounds area after 0 (wound area = 100%), 24, 48 and 72 h.

#### Immunocytochemistry (ICC) to illustrate ß-catenin shift

Subcellular localization of ß-catenin while adding Wnt signaling manipulators was investigated by ICC. After A2780 and A2780cis cells grew on microscope slides to subconfluency, they were incubated with 100 µM SB216763 or 50 µM XAV939 for 72 h. The slides were fixed putting them into phosphate-buffered saline (Gibco) for 5 min and methanol for 5 min, followed by freezing at − 20 °C. Manufactured slides were treated with anti-β-catenin IgG (Diagnostic BioSystems, Pleasanton, CA, USA) (Table 1) overnight in a moist chamber. Thereafter Vectastain Elite rabbit-IgG-Kit (Vector Laboratories, Burlingame, CA, USA) was used to detect and visualize ß-catenin by the ABC-method with 3-amino-9-ethylcarbazole (AEC) as chromogenic substrate. Slides were counterstained with hemalaun. The images were captured using a microscope including a digital camera system (Carl Zeiss, Jena, Germany). Controls of cells treated with 2‰ (for incubation with SB216763) and 5‰ (for incubation with XAV939) DMSO were carried out.

#### Statistical analysis of cell culture experiments

Statistical analysis was performed with GraphPad Prism 8 (GraphPad Software, La Jolla, CA, USA). Significant differences to controls were determined by one-way ANOVA followed by Dunnett's multiple comparisons test or two-way ANOVA followed by Sidak's multiple comparisons test. A probability of *p* < 0.05 was considered significant. The columns in each graph show the mean of relative values in % or as an enrichment factor. Therefore, the presentation of error bars is not applicable in our study.

### Experiments on human tissue samples

#### Patient cohort and ethics approval

Specimens represent a cohort of 153 patients with EOC (serous [*n* = 109], endometrioid [*n* = 21], clear cell [*n* = 11], mucinous [*n* = 12]) who underwent radical cytoreductive surgery in our department between 1990 and 2002. Histopathological diagnoses were established by a specialized gynecologic pathologist with staging and grading according to TNM and FIGO (WHO) classification. 75.2% of patients presented with advanced disease (FIGO IIB-IV), while only 24.8% were diagnosed in early disease (FIGO I-IIA). Except for patients in stage FIGO IA with low-grade histology, all patients received adjuvant platinum-based chemotherapy. Lifetime data (birth, primary OC diagnosis, relapse, death) from EOC patients were taken from our patient charts, the Munich Cancer Registry and aftercare calendars. Median age at primary diagnosis was 59.0 years with a 95% confidence interval (CI) of 57.0–61.0 years. 28 relapses and 101 deaths were documented. A summary of patient characteristics can be found in Table 2. Our study has been approved by the ethics committee of Ludwig Maximilian University of Munich (reference number 138/03) and was carried out in compliance with the guidelines of the Helsinki Declaration of 1964 (last revision October 2018). All participants gave their written informed consent. Samples and clinical information were anonymized for statistical workup.

**Table 2:** Patient characteristics: shown are the categorization for histological subtype, grading and FIGO stage of the specimens from the cohort of 153 patients with EOC and a summary of their according lifetime data

Histology and stage				
	Category		*n*	%
Subtype and grading	Serous	Low-grade	24	15.7
		High-grade	80	52.3
		Not classified	5	3.3
		Total	109	71.3
	Endometrioid	G1	6	3.9
		G2	5	3.3
		G3	8	5.2
		Not classified	2	1.3
		Total	21	13.7
	Clear cell	G3	9	5.9
		Not classified	2	1.3
		Total	11	7.2
	Mucinous	G1	6	3.9
		G2	6	3.9
		G3	0	0.0
		Not classified	0	0.0
		Total	12	7.8
FIGO	I		35	22.9
	II		12	7.8
	III		103	67.3
	IV		3	2.0

Lifetime data				
	Median (years)		95% CI (years)	
Age at diagnosis	59.0		56.0–60.0	
OS	3.6		2.0–5.3	

#### Tissue microarray (TMA)

Out of representative regions of the paraffin-embedded tumor samples biopsies 0.6 mm in diameter were taken and arrayed into a recipient paraffin block (30 × 20 × 10mm) using a microtissue arrayer (Beecher Instruments, Sun Prairie, WI, USA). Every tumor sample was used for three biopsies, resulting in 459 TMAs in total. Afterwards, sections of 5 µm were prepared and transferred to microscope slides. To determine whether there was enough representative tumor tissue left, a haematoxylin and eosin stain was done.

#### Immunohistochemistry (IHC) for TMAs

IHC was performed using a combination of pressure cooker heating and the ZytoChem-Plus HRP Polymer-Kit with DAB as chromogenic substrate according to a previous publication by our lab (Scholz et al. 2012). The primary antibodies in our immunohistochemical staining were anti-β-catenin IgG, anti-E-cadherin IgG (Merck, Darmstadt, Germany) and anti-snail/ slug IgG (Abcam, Cambridge, UK) (Table 1). Evaluation, imaging and storing was done with an AxioScope microscope (Carl Zeiss), an AxioCam digital camera system (Carl Zeiss) and the AxioVision software (Carl Zeiss). Immunohistochemical staining was assessed semiquantitatively, according to Remmele and Steger (Remmele and Stegner 1987) using the IHC score (mean ± SEM). Expression of Wnt signaling markers was captured in different subcellular locations (β-catenin: membrane and plasma, snail/ slug: plasma, E-cadherin: membrane and plasma).

#### Statistical analysis of tissue sample experiments

Mean values of the three representative IHC scores of every probe were calculated for further analysis. GraphPad Prism 8 was used for the comparison of immunoquantitation between histological subtypes, FIGO stages and platinum-response (< 6 months to primary surgery)/ -sensitive (≥ 6 month to primary surgery) with Mann–Whitney-*U* test (mean ± SEM). Furthermore, expression-dependent differences in overall survival (OS) (median ± SEM) and progression-free survival (PFS) (median ± SEM) were tested by chi-square statistic of the Log-Rank test (Mantel-Cox) in Kaplan–Meier curves with SPSS Statistics 25 (IBM, Chicago, IL, USA). *P*-values < 0.05 were considered to be statistically significant for further analyses.

## Results

### In vitro experiments with cultured cell lines

#### Activated Wnt signaling in platinum-resistant EOC cells can be reversed by Wnt manipulators

**Fig. 2:**
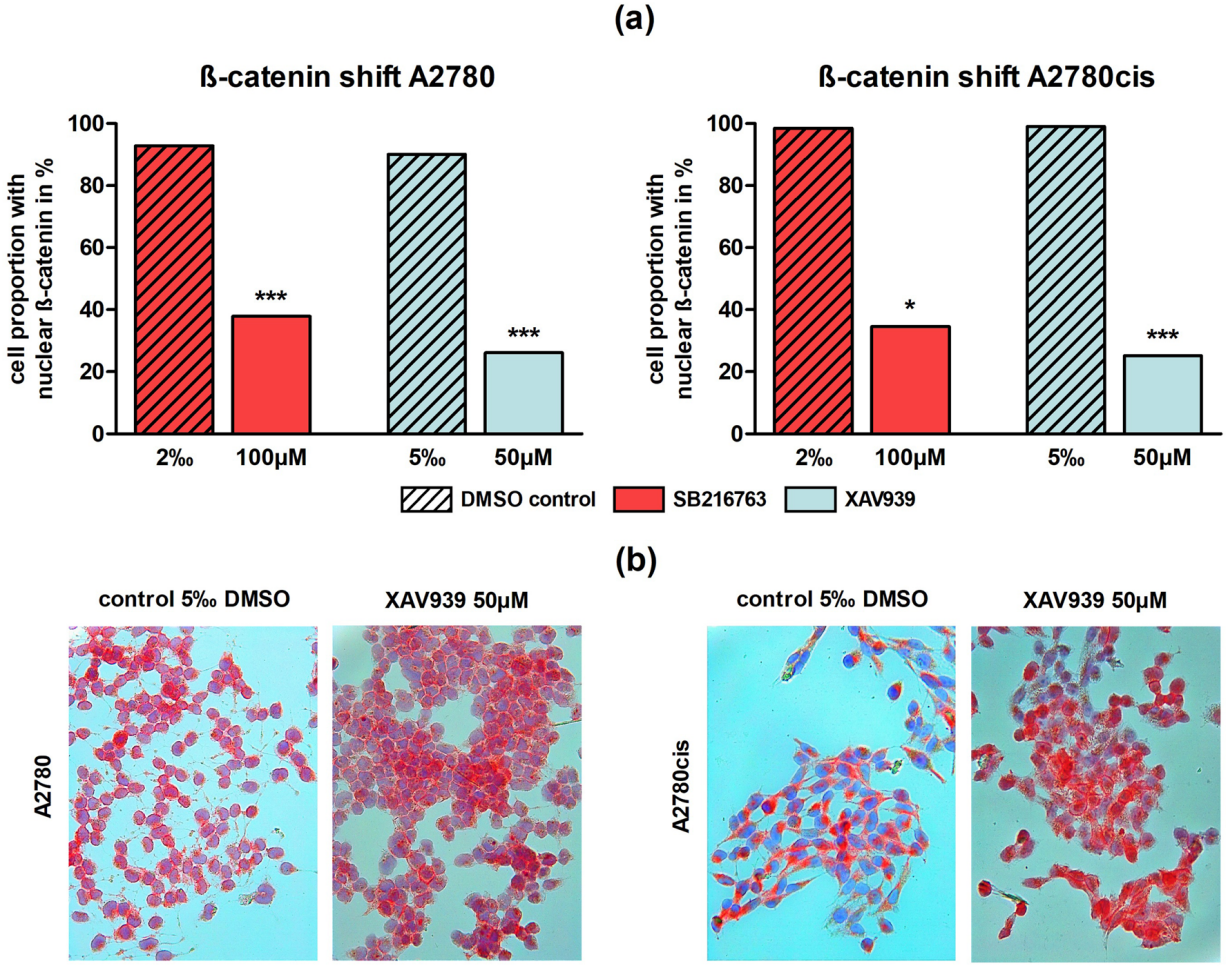
ß-catenin shift after treatment with different Wnt signaling manipulators in A2780 and A2780cis: **a** In A2780 and A2780cis the proportion of cells with nuclear localization of ß-catenin was significantly decreased through the addition of 100 µM SB216763 or 50 µM XAV939 compared to controls (*n* = 3 per column, mean, *p < 0.05/ ***p < 0.001 by Sidak's multiple comparisons test). **b** Representative images of A2780 and A2780cis after treatment with 50 µM XAV939 compared to controls with 5‰ DMSO. ß-catenin is red-colored. While in controls ß-catenin is mainly localized in the nucleus, addition of the Wnt inhibitor XAV939 leads to a shift towards the cell membrane

ICC of ß-catenin was performed to indicate activation of the canonical Wnt pathway according to its nuclear localization. A significant (*p* < 0.05 or *p* < 0.001) shift from ß-catenin staining from the nucleus to the membrane was noted following SB216763 (100 µM) and XAV939 (50 µM) treatment in comparison to controls for both cell lines A2780 and A2780cis. The biggest impact presented XAV939 treatment in A2780cis, where the cell proportion with nuclear ß-catenin localization decreased from 99.1% (control with 5‰ DMSO) to 25.2% (*p* < 0.001) (Fig. 2).

#### Wnt manipulators and their effect on proliferation

**Fig. 3:**
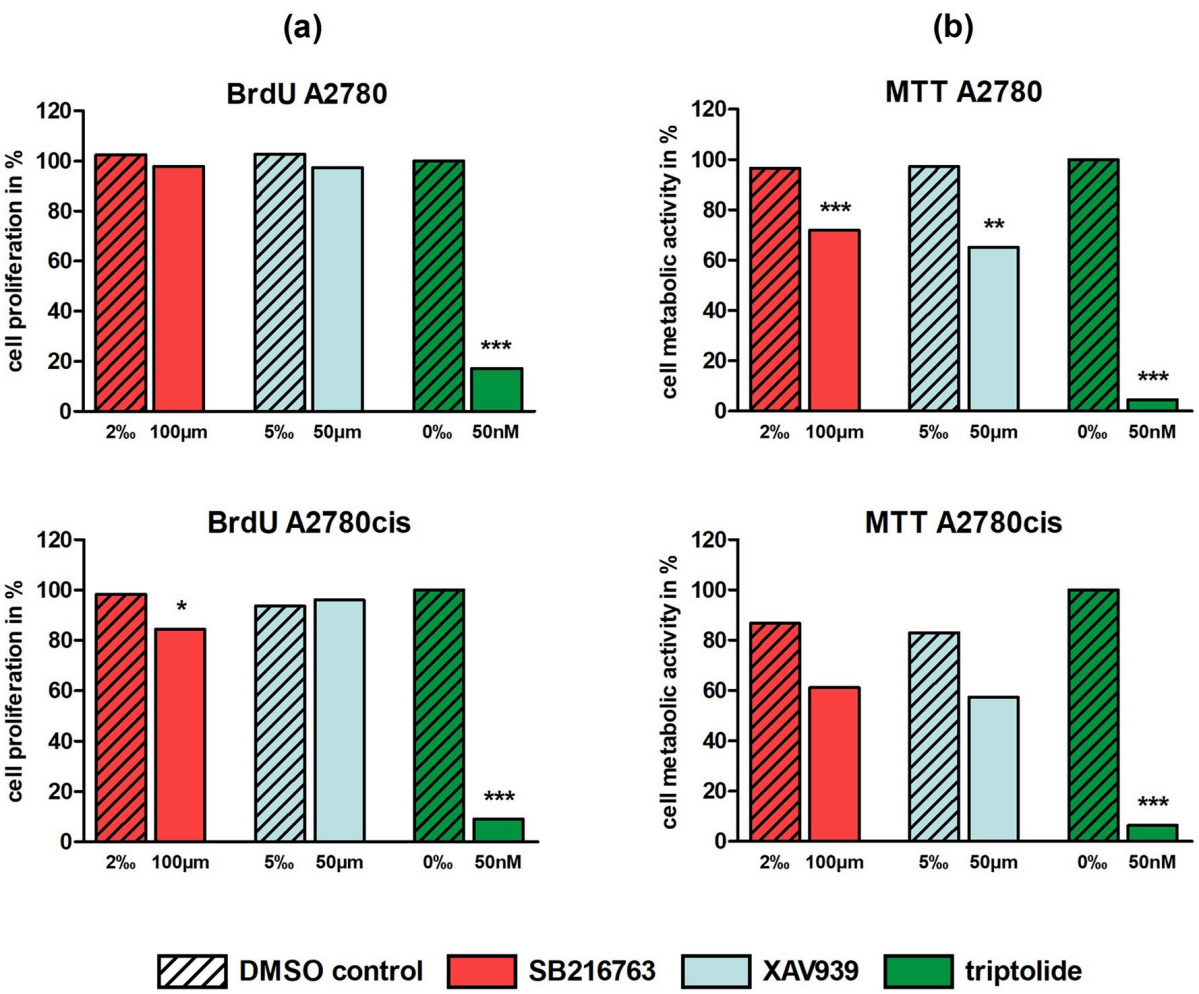
BrdU cell proliferation and MTT cell viability assay for different Wnt signaling manipulators (100 µM SB216763, 50 µM XAV939, 50 nM triptolide) in A2780 and A2780cis after 72 h: **a** In the BrdU assay only triptolide displayed a significant impairment of proliferation in A2780 and A2780cis, whereas SB216763 revealed a significant reduction of cell proliferation in A2780cis and XAV939 showed no influence (*n* = 9 per column, mean, *p < 0.05/ ***p < 0.001 by Dunnett's multiple comparisons test, controls with 0‰ DMSO were set 100%). **b** In the MTT assay all three inhibitors in platinum-sensitive and -resistant cells reduced metabolic activity with different levels of significance (*n* = 9 per column, mean, **p < 0.01/ ***p < 0.001 by Dunnett's multiple comparisons test, controls with 0‰ DMSO were set 100%)

To evaluate the effect on proliferation of EOC cell lines following treatment with Wnt manipulators, BrdU assay was carried out. Since the inhibition of cell proliferation showed a dose-dependent positive correlation for triptolide but not for SB216763 or XAV939 (Supp. 1a), we compared the highest concentrations of each inhibitor against its control. Controls for triptolide without DMSO were set 100.0%. After 72 h, triptolide (50 nM) displayed a significant (*p* < 0.001) impairment of proliferation in A2780 (17.1%) and A2780cis (8.9%). While XAV939 (50 µM) showed no influence in both cell lines, SB216763 (100 µM) also significantly (*p* < 0.05) inhibited proliferation in A2780cis (84.5%) (Fig. 3a).

#### Impaired cell viability by Wnt manipulators

Cell viability was examined with the MTT assay. In accordance with the BrdU assay, a dose dependence for the concentrations tested, was found only for triptolide (Supp. 1b). But in contrast to cell proliferation assay, metabolic activity decreased for all three inhibitors in platinum-sensitive (SB216763: 72.0%, XAV939: 65.2%, triptolide: 4.7%) and -resistant cells (SB216763: 61.2%, XAV939: 57.3%, triptolide: 6.4%) in MTT assay. Except for SB216763 and XAV939 in A2780cis, these findings were highly significant (*p* < 0.01 or *p* < 0.001) at the selected concentrations of 100 µM SB216763, 50 µM XAV939 and 50 nM triptolide (Fig. 3b). While triptolide already led to significantly reduced metabolic activity after 48 h, this effect was seen for the other agents not before 72 h.

#### Enhanced apoptosis by triptolide in a dose-dependent manner

**Fig. 4:**
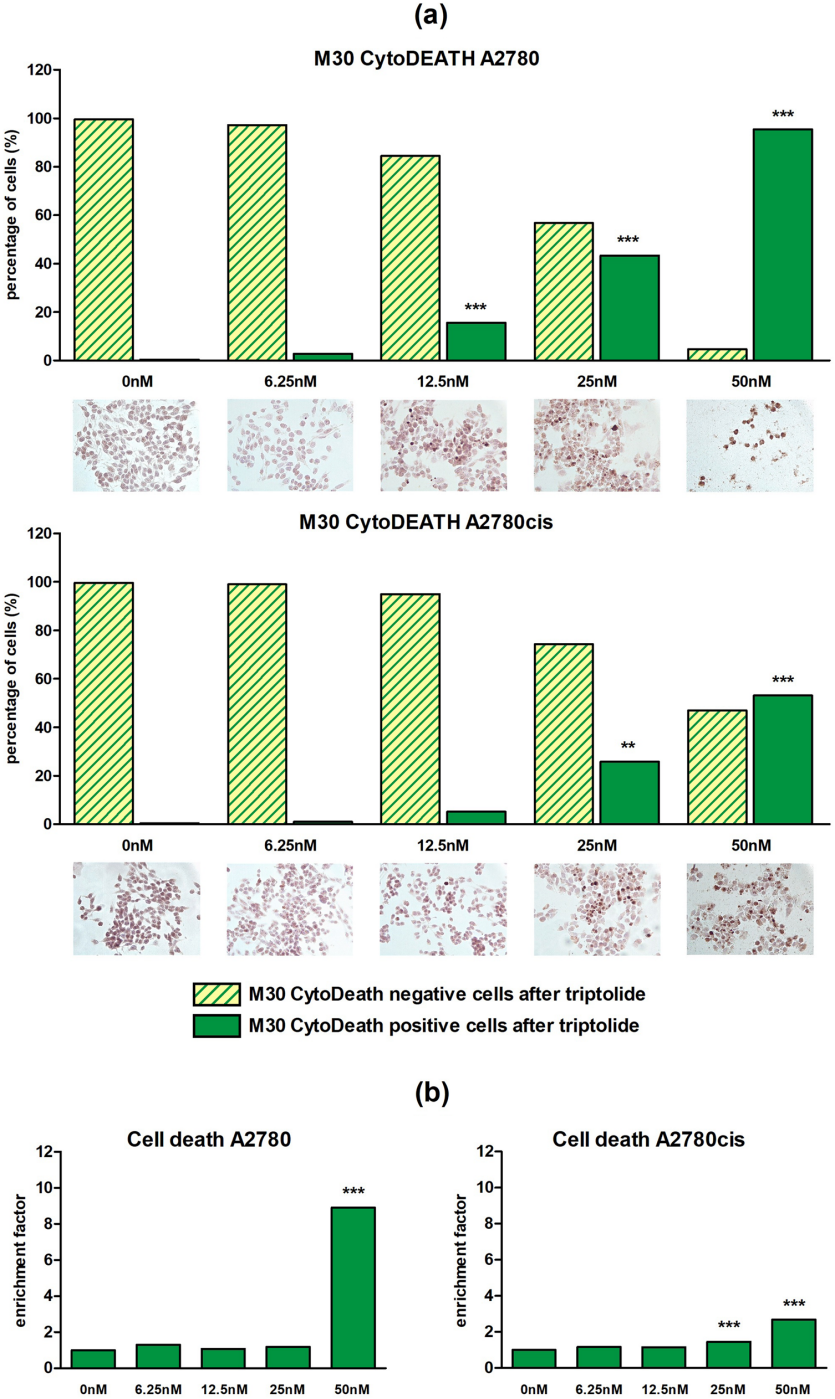
CytoDEATH apoptosis assay after 48 h and Cell death detection ELISA after 24 h for different triptolide concentrations in A2780 and A2780cis: **a** Apoptosis of cell lines A2780 and A2780cis was enhanced by triptolide in a dose-dependent manner. The percental rate of apoptic cells (M30 CytoDEATH positive) significantly increased in both cell lines with rising concentrations. Meanwhile, M30 CytoDEATH negative cells rapidly decreased (*n* = 3 per column, mean, **p < 0.01/ ***p < 0.001 by Dunnett's multiple comparisons test). According to documented concentrations representative microphotographs of EOC cells with an apoptic cell proportion in intense brown after DAB treatment are added. **b** Incubation for 24 h with different triptolide concentrations revealed a dose-dependent rise of absorbance up to a maximum at 50 nM, which confirms enhancement of apoptosis by triptolide (*n* = 6 per column, mean, ***p < 0.001 by Dunnett's multiple comparisons test, controls without DMSO were set 1)

To determine a Wnt inhibition induced early apoptosis, triptolide was chosen for M30 CytoDEATH apoptosis assay due to its strong impact in the previous experiments. Following 48 h of treatment with 6.25 nM, 12.5 nM, 25 nM or 50 nM triptolide, the percentage of apoptotic cells (M30 CytoDEATH positive) increased significantly (p < 0.01 or *p* < 0.001) in a dose-dependent manner up to 95.4% for A2780 and to 53.1% for A2780cis, respectively. Controls with 0‰ DMSO showed 100.0% M30 CytoDEATH negative cells (Fig. 4a).

Moreover, histone-associated DNA-fragments, produced as a result of apoptosis, were quantified using a cell death detection ELISA. Incubation for 24 h with identical triptolide concentrations showed a highly significant (*p* < 0.001) dose-dependent rise of absorbance up to 8.9-times in A2780 and 2.7-times in A2780cis at 50 nM (Fig. 4b), confirming apoptosis' dose-dependence from triptolide in both cell lines.

#### Cell migration is inhibited by SB216763 and triptolide

**Fig. 5:**
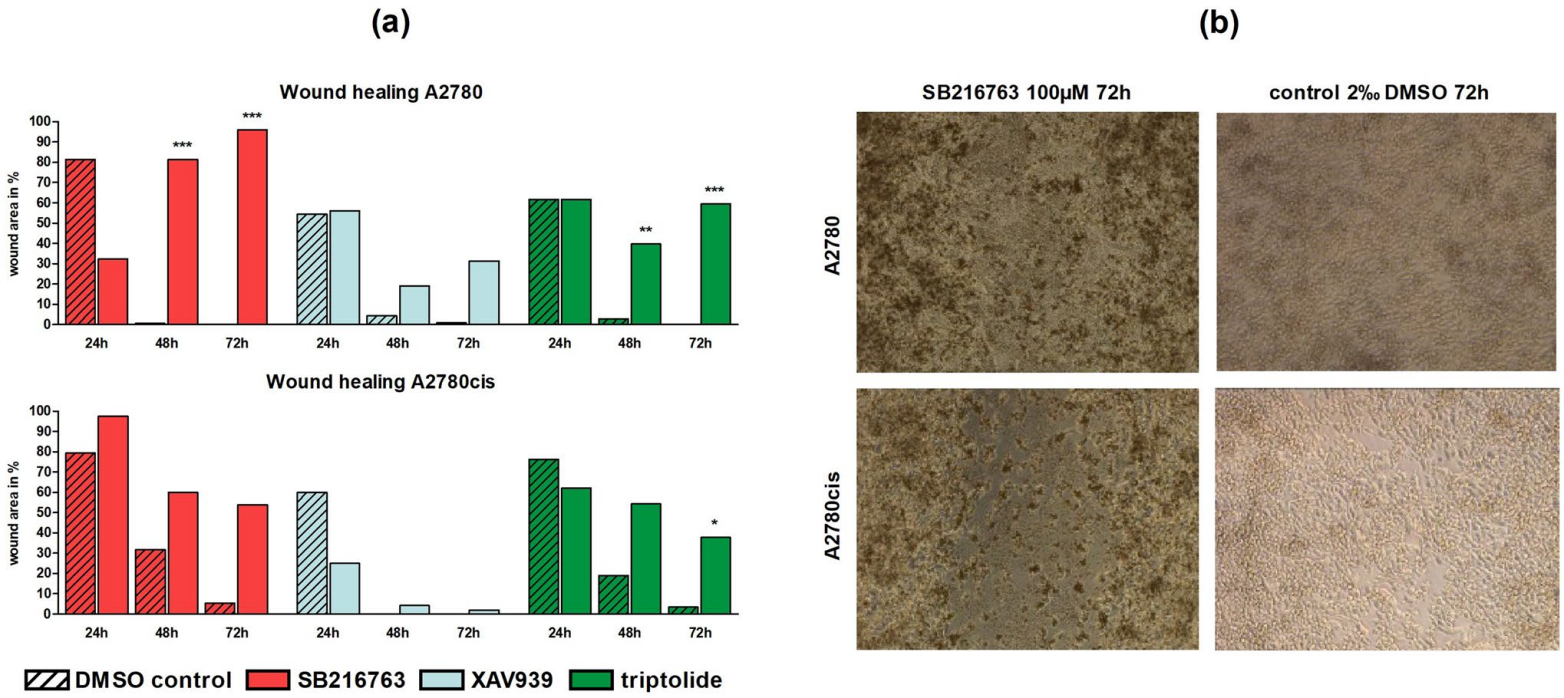
Wound healing assay for different Wnt signaling manipulators in A2780 and A2780cis: **a** Cell migration in A2780 and A2780cis was significantly reduced through the addition of 100 µM SB216763 or 50 nM triptolide after 48 and 72 h compared to controls. Treatment with XAV939 did not affect migration ability at all (*n* = 3 per column, mean, *p < 0.05/ **p < 0.01/ ***p < 0.001 by Sidak's multiple comparisons test, wound area after 0 h was set 100%). **b** Representative images of the wound area in A2780 and A2780cis 72 h after treatment with 100 µM SB216763 compared to controls with 2‰ DMSO. While controls showed a confluent monolayer of cells, in SB216763 treated cells a remaining wound area is visible

Migration capacity of OC cells was monitored within vitro wound healing assays with 100 µM SB216763, 50 µM XAV939 or 50 nM triptolide. In A2780, SB216763 and triptolide led to a significant reduction in wound healing after 48 and 72 h (*p* < 0.01 or *p* < 0.001) (Fig. 5a). After 72 h, the remaining wound area for SB216763 was 95.9% (Fig. 5b) and 59.5% confluent for triptolide compared to 0.0% in both controls. Treatment with XAV939 whereas did not affect migration ability in A2780 and A2780cis. In the platinum-resistant cell line, we saw the identical but slightly lower impact of SB216763 (Fig. 5b) and triptolide. Following treatment with 50 nM triptolide after 72 h the remaining wound area was 37.8% compared to 3.3% in the control (Fig. 5a).

### Results on human tissue samples

#### Markers for Wnt signaling with different expression patterns in serous compared to other subtypes

To understand the role of Wnt pathway in the clinical context, immunoquantitative comparisons of Wnt signaling markers among four histological subtypes (serous, endometrioid, clear cell, mucinous) were performed. IHC score revealed multiple significant expression differences especially of serous histology compared to other subtypes (Table 3). Membranous and plasmatic ß-catenin expression was significantly higher (*p* < 0.001) in endometrioid (membrane: 10.2 ± 0.4, plasma: 10.0 ± 0.4), clear cell (membrane: 10.1 ± 0.5, plasma: 10.2 ± 0.4) and mucinous (membrane: 11.2 ± 0.4, plasma: 10.4 ± 0.8) type compared to serous (membrane: 7.6 ± 0.2, plasma: 6.4 ± 0.2) histology. Moreover, in comparison to serous subtype, expression of plasmatic snail/ slug (8.8 ± 0.2) and membranous E-cadherin (7.6 ± 0.3) was significantly lower in endometrioid (snail/ slug: 7.2 ± 0.5 [p < 0.05], E-cadherin: 4.7 ± 0.9 [*p* < 0.001]) and clear cell (snail/ slug: 5.4 ± 0.5 [*p* < 0.001], E-cadherin: 5.0 ± 1.0 [*p* < 0.05]) histology. Representative stainings are displayed in Fig. 7b.

**Table 3:** Comparison of IHC scores of Wnt signaling markers between histological subtypes: IHC scores in TMAs for β-catenin, snail/ slug and E-cadherin in different subcellular locations were compared between four different types of EOC (serous [*n* = 109], endometrioid [*n* = 21], clear cell [*n* = 11], mucinous [*n* = 12])

	Serous vs. endometrioid		Serous vs. clear cell		Serous vs. mucinous	
β-Catenin (membrane)	7.6 ± 0.2	10.2 ± 0.4	7.6 ± 0.2	10.1 ± 0.5	7.6 ± 0.2	11.2 ± 0.4
	< ***	< ***	< ***	< ***	< ***	< ***
β-Catenin (plasma)	6.4 ± 0.2	10.0 ± 0.4	6.4 ± 0.2	10.2 ± 0.4	6.4 ± 0.2	10.4 ± 0.8
	< ***	< ***	< ***	< ***	< ***	< ***
snail/ slug (plasma)	8.8 ± 0.2	7.2 ± 0.5	8.8 ± 0.2	5.4 ± 0.5	8.8 ± 0.2	6.9 ± 1.1
	> *	> *	> ***	> ***	n.s	n.s
E-Cadherin (membrane)	7.6 ± 0.3	4.7 ± 0.9	7.6 ± 0.3	5.0 ± 1.0	7.6 ± 0.3	7.4 ± 1.1
	> ***	> ***	> *	> *	n.s	n.s
E-Cadherin (plasma)	7.4 ± 0.2	8.0 ± 0.7	7.4 ± 0.2	5.4 ± 0.9	7.4 ± 0.2	8.5 ± 0.9
	n.s	n.s	> *	> *	n.s	n.s

Analysis revealed significant (mean ± SEM, *p < 0.05/ ***p < 0.001 by Mann–Whitney-*U* test) and non-significant results (*n.s.*)

#### ß-catenin and E-cadherin show significant expression differences with regard to FIGO stage

**Fig. 6:**
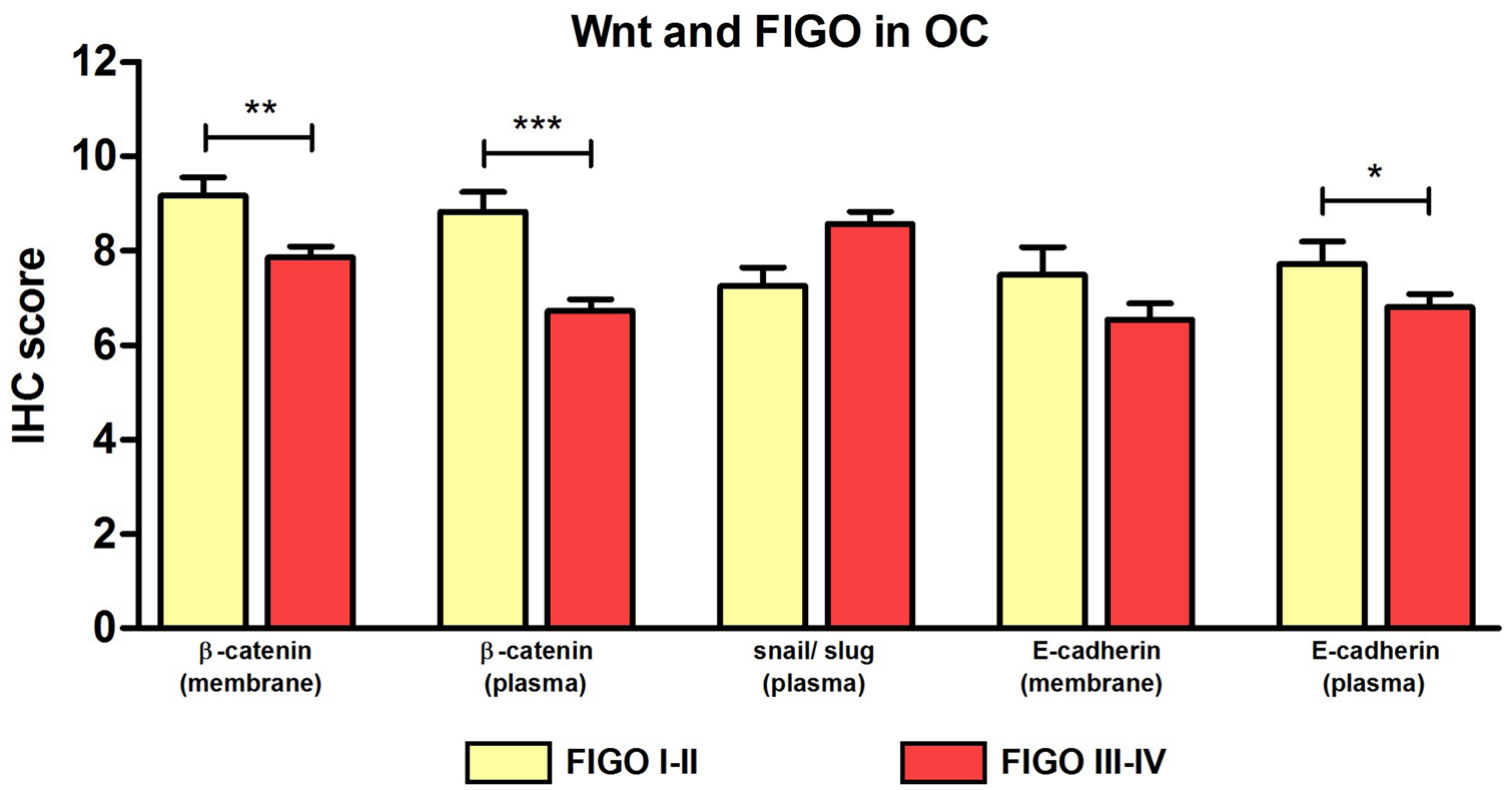
Comparison of IHC scores of Wnt signaling markers dependent on FIGO stages: Analyzing expression of Wnt signaling markers dependent on FIGO stages (*n* = 153) revealed significant differences for β-catenin and E-cadherin in two different locations (membrane and plasma) (mean ± SEM, *p < 0.05/ **p < 0.01/ ***p < 0.001 by Mann–Whitney-*U* test)

Comparing expression of Wnt markers with regard to FIGO stage, significantly higher expression of ß-catenin (membrane) (9.2 ± 0.4 to 7.9 ± 0.2 [*p* < 0.01]), ß-catenin (plasma) (8.8 ± 0.4 to 6.7 ± 0.3 [*p* < 0.001]) and E-cadherin (plasma) (7.7 ± 0.5 to 6.8 ± 0.3 [*p* < 0.05]) were noted for FIGO stage I-II compared to FIGO stage III-IV (Fig. 6).

No significant expression differences were identified for Wnt markers in platinum-resistant compared to platinum-sensitive patients (data not shown).

#### Higher plasmatic snail/ slug expression is associated with significantly longer PFS in serous OC

**Fig. 7:**
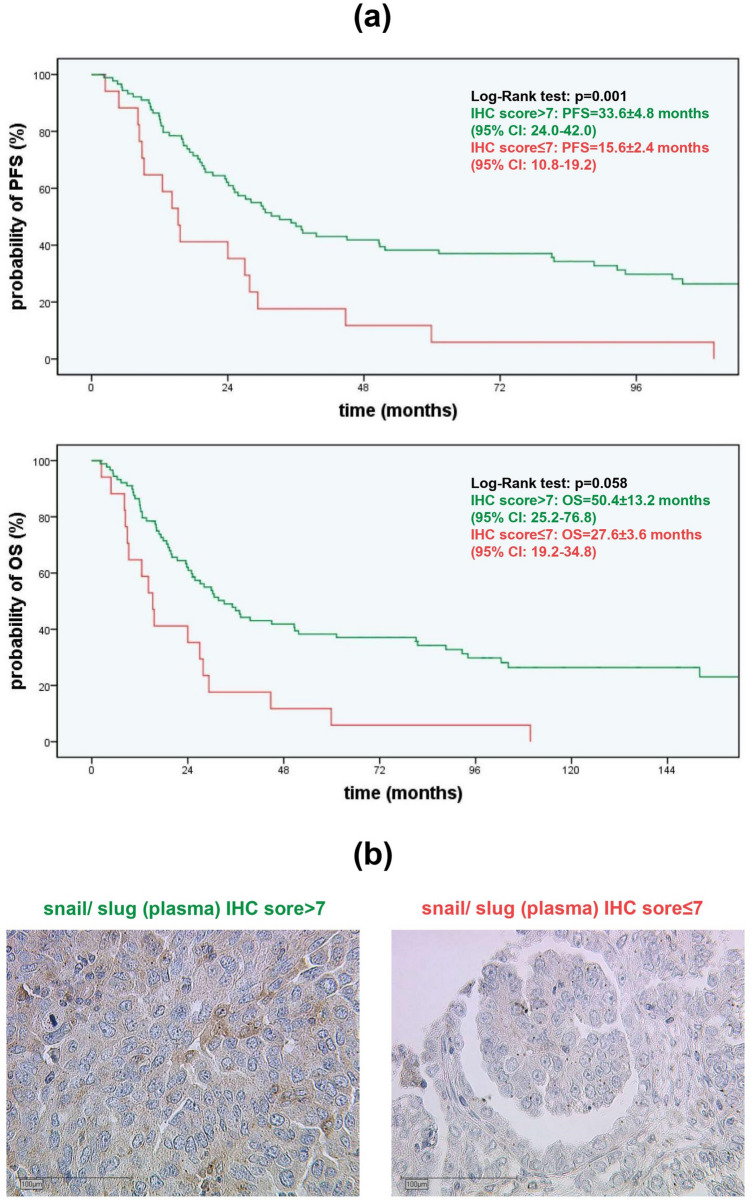
PFS depending on plasmatic snail/ slug IHC scores in serous EOC and representative microphotographies: **a** The Kaplan–Meier curves demonstrate PFS and OS as a function of plasmatic snail/ slug expression in serous OC cases. Green lines (IHC score > 7, PFS = 33.6 ± 4.8 months, OS = 50.4 ± 13.2 months) show a significant benefit in PFS and a trend in OS compared to the red line (IHC score ≤ 7, PFS = 15.6 ± 2.4 months, OS = 27.6 ± 3.6 months) (median ± SEM, p = 0.001/ p = 0.058 by chi-square statistic of the Log-Rank test [Mantel-Cox]). **b** Plasmatic snail/ slug accumulation in serous subtype is marked in brown after DAB treatment. While the left microphotography shows a higher expression (IHC score > 7), on the right photography a lower expression (IHC score ≤ 7) is shown

Prognostic impact of Wnt pathway markers on OS and PFS were tested for the whole cohort and for each subtype. For serous subtype, a significantly longer PFS was noted for higher plasmatic expression of snail/ slug (plasma) expression with a cut-off IHC score of seven or beneath (33.6 ± 4.8 to 15.6 ± 2.4 months [*p* = 0.001]). A similar trend was noted for OS, although this difference did not reach statistical significance (50.4 ± 13.2 to 27.6 ± 3.6 months [*p* = 0.058]) (Fig. 7).

## Discussion

Development of platinum-resistance is one of the major challenges in the clinical management of EOC. Despite recent advances with the inclusion of targeted therapies to standard treatment as the anti-angiogenic bevacizumab (Burger et al. 2011; Perren et al. 2011) or PARP inhibitors (Coleman et al. 2017; Ledermann et al. 2012, 2014; Mirza et al. 2016; Pujade-Lauraine et al. 2017), no real improvements to overcome resistance to platinum in the clinical course have been achieved so far. Since different studies suggest that manipulation of the Wnt pathway may have an impact on tumor progression (Barghout et al. 2015; Boone et al. 2016; Cao et al. 2006; Li et al. 2016; Nagaraj et al. 2015; Rivard et al. 2014; Westfall et al. 2008), we identified three molecules as promising examples for manipulators of the Wnt pathway to investigate the opportunity of resensitization of EOC cells to platinum.

During application of SB216763, XAV939 and triptolide to EOC cell lines A2780 and A2780cis in functional tests in vitro, only triptolide exhibited a continuous effect on cell biology with a significant reduction of cell proliferation and cell viability as well as induction of apoptosis. Our results confirm the existing in vitro and in vivo studies in OC cells (Rivard et al. 2014; Westfall et al. 2008): triptolide is able to reduce ovarian tumor cell progress applied as a single agent or compared with carboplatin. But in contrast to earlier studies, we directly used triptolide but no prodrug.

The inhibitor of canonical Wnt signaling XAV939 led to significantly impaired cell viability matching with a study from Li et al., in which the importance of long non-coding RNA HOTAIR for Wnt signaling driven chemoresistence of OC cells was demonstrated and XAV939 was able to partially block this effect (Li et al. 2016). Furthermore, in colorectal cancer cells XAV939 significantly increased the apoptotic cell fraction alone or in combination with 5-fluorouracil and cisplatin (Wu et al. 2016). Indeed, experiments on XAV939′s direct impact on both platinum-resistant and -sensitive EOC cells were missing by now, as well as for SB216763.

Interestingly, treatment with this GSK-3β inhibitor and thus Wnt activator presented similar results as for XAV939 treatment. In addition, SB216763 treatment resulted in a significant reduction of cell proliferation in A2780cis. Our results are consistent with the work of Cao et al. and Schulz et al., who detected GSK-3β as a driving force in tumor cell progression in EOC (Cao et al. 2006) and squamous cell carcinomas of the head and neck (Schulz et al. 2018). These results confirm that GSK-3β necessarily influences functional tests on EOC cells via other pathways besides Wnt, like a NF-κB-dependent pathway (Ougolkov et al. 2005) or a modulation by the tumor microenvironment (Fridman et al. 2014; Giraldo et al. 2019). Furthermore, the diversity of Wnt's target gene panel (Arend et al. 2013; Talbot et al. 2012) also offers options for inhibitory functions of Wnt in tumor progression.

Another finding of this study was the ability of all three drugs tested, to inhibit cell migration. Significance was seen for triptolide in both cell lines as well as for SB216763 in A2780. Epithelial-mesenchymal transition (EMT) is a key mechanism in cell migration and Wnt signaling is one of EMT's major pathways (Talbot et al. 2012). In OC, the inhibition of EMT by treatment with a Wnt repressor (salinomycin) has been proved already (Li et al. 2017), but to our knowledge this is the first data set on a platinum-resistant cell line. Concordant with the putative bipolarity of GSK-3β in tumor biology, results on the transcriptional profile of various EMT related genes in response to SB216763 were summarized as a dysregulated EMT without any clear direction by the authors of this study (Schulz et al. 2018).

Summarizing the functional tests mentioned above SB216763, XAV939 and triptolide effect both platinum-sensitive and -resistant cells. The results provide the opportunity to potentiate the impact of a platinum-based chemotherapy but were not able to show a fully resensitization of EOC cells to carboplatin. This confirms findings on other Wnt inhibitors in OC (Barghout et al. 2015; Boone et al. 2016; Nagaraj et al. 2015; Rivard et al. 2014; Westfall et al. 2008) and opens promising perspectives for clinical management of platinum-resistant patients.

Of course, varieties in significance levels throughout the functional tests are a limitation of this study and most likely caused by relatively small sample sizes. Nevertheless, this was not the primary aim of our work and needs further examinations. Moreover, the cell line A2780 mainly represents features of the endometrioid subtype (Anglesio et al. 2013; Köbel et al. 2008) and is thus not able to act as a reliable model for all histological subtypes of EOC. However, especially in OC research it is a well-established cell model, representing the typical contrast of platinum-resistant and -sensible cells.

To improve the understanding of Wnt signaling’s diverse role in OC progress, we aimed to detect the localization of β-catenin (membrane versus nucleus) after SB216763 or XAV939 treatment in both cell lines. In parallel to the functional tests, both drugs led to an immunohistochemical shift of β-catenin from the nucleus (Wnt signaling activated) to the membrane (Wnt signaling inactivated), which finally contrasts the general role of SB216763 as an activator of the pathway (Naujok et al. 2014). Our results are in line with a diverse role of GSK-3β in cancer's context (Patel and Woodgett 2008) and may support a general dysregulation of Wnt and EMT in some cancers (Schulz et al. 2018). Certainly, the phosphorylation pattern is one approach to explain this bipolarity. In general, the kinase is inactivated through phosphorylation at serine residue nine (GSK-3β[pS9]) or activated through phosphorylation at tyrosine residue 216 (GSK-3β[pY216]) (Domoto et al. 2016; Fang et al. 2002). Paradoxically, a completely deregulated activity of GSK-3β according to modifications in the differential phosphorylation of S9 and Y216 residues was seen in gastrointestinal cancers (Mai et al. 2009) and glioblastoma (Miyashita et al. 2009) compared to "healthy" cells. The present results support this thesis in OC for the first time.

To correlate the in vivo examinations with the impact of Wnt marker proteins on the clinical course of OC, a homogenously treated cohort of formalin-fixed paraffin-embedded tissue from EOC patients was examined. In a comparison between the histologic subtypes, the serous subtype is usually thought to have the highest activity of Wnt signaling (Lee et al. 2003), as it is dominated by the high-grade carcinomas with their poor prognosis (Jayson et al. 2014; Köbel et al. 2008). Our study supports this observation, since the small fraction of extranuclear β-catenin in serous subtype compared to the others as well as in FIGO stage III-IV compared to FIGO stage I-II.

E-cadherin, a molecule mediating cell–cell adhesion, is usually regarded as an invasion suppressor. However, especially EOC cells additionally undergo a mesenchymal-epithelial transition. Thus, in contrast to other cancers (Berx and van Roy 2009), E-cadherin is mostly elevated. The higher E-cadherin expression in lower stages could be confirmed as previously described (Arend et al. 2013; Bodnar et al. 2014), but we detected a significant difference in E-cadherin expression between the different subtypes.

Snail/ slug is a transcription factor, localized in the plasma of the cell when Wnt signaling is inactivated (Kim et al. 2012). Interestingly, it is the only Wnt marker tested with a clear correlation to PFS or OS: in our study an elevated expression of snail/ slug in the serous subtype is significantly associated with longer PFS and shows a positive trend for OS. The transcription factor snail/ slug is well-known for its tumor-promoting influence as a driver in EMT. Induced autophagy of this protein led to control of EMT and metastasis in a HeLa cell model (Zada et al. 2019) and knockdown of it suppresses ovarian tumor growth (Baldwin et al. 2014). In lung carcinoma cells this positive influence on tumor progress was clearly correlated to translocation into the nucleus (Perumal et al. 2019). Since current scientific data for snail/ slug’s role are homogenous among each other and with our results, inhibition of snail/ slug or at least persistent shift towards plasma might be a promising base for prognostic approaches in the future.

## Conclusion

Functional tests investigating the impact of the Wnt manipulators SB216763, XAV939 and triptolide on the OC cell lines A2780 and A2780cis detected significant effects on both platinum-sensitive and -resistant cells. With this, all three manipulators provide the opportunity to emphasize the impact of a platinum-based chemotherapy. While specific results of the substances were heterogenous, the inhibitory impact of triptolide itself on OC tumor progression and its promoting impact on apoptosis has to be highlighted. Repression of EMT markers by Wnt inhibition was shown for the first time in the context of platinum-resistant OC cell lines. Our analysis on the existing EOC patient cohort confirmed a potential role of the Wnt pathway in serous OC cases, which is of note as it is the most common histological subgroup of OC. Together with the different expression levels of β-catenin and E-cadherin between FIGO stages and the impact of the expression levels for snail/ slug on prognosis, this study enables perspectives for clinical management of platinum-resistant patients through manipulation of the Wnt/ β-catenin pathway.

## Electronic supplementary material

Below is the link to the electronic supplementary material.

Supplementary file1 (JPG 678 kb)

Supp. 1 BrdU cell proliferation and MTT cell viability assay for different Wnt signaling manipulators in different concentrations in A2780 and A2780cis after 72h: (a) In the BrdU assay only triptolide displayed a dose-dependent decrease of cell proliferation in A2780 and A2780cis, whereas SB216763 and XAV939 showed no dose-dependency (n=9 per column, mean, controls with 0‰ DMSO were set 100 %). (b) In the MTT assay, according to the findings in the BrdU assay, only triptolide displayed a dose-dependent decrease of cell metabolic activity in A2780 and A2780cis, whereas SB216763 and XAV939 showed no dose-dependency (n=3 per column, mean, controls with 0‰ DMSO were set 100 %)

## Data Availability

The datasets generated and analysed during the current study are available from the corresponding author on reasonable request.

## Abbreviations

AEC: 3-Amino-9-ethylcarbazole
DAB: 3,3′-Diaminobenzidine
MTT: 3-(4,5-Dimethylthiazol-2-yl)-2,5-diphenyltetrazolium bromide
BrdU: 5-Bromo-2′-deoxyuridine
APC: Adenomatous polyposis coli
CI: Confidence interval
DMSO: Dimethyl sulfoxide
Dsh: Dishevelled
ELISA: Enzyme-linked immunosorbent assay
EMT: Epithelial-mesenchymal transition
EOC: Epithelial ovarian cancer
FZD: Frizzled
FIGO: International Federation of Gynecology and Obstetrics
ICC: Immunocytochemistry
IHC: Immunohistochemistry
LRP: Lipoprotein receptor-related protein
LEF: Lymphoid enhancer factor
OC: Ovarian cancer
OS: Overall survival
PARP: Poly ADP ribose polymerase
PFS: Progression-free survival
TCF: T-cell factor
TMA: Tissue microarray
Ub: Biquitination
