# Supplementary figures and images for: Potential of platinum-resensitization by Wnt signaling modulators as treatment approach for epithelial ovarian cancer
Source: J Cancer Res Clin Oncol. 2020 Jul 17;146(10):2559–74. doi: 10.1007/s00432-020-03317-4 (PMC7467966; doi:10.1007/s00432-020-03317-4)

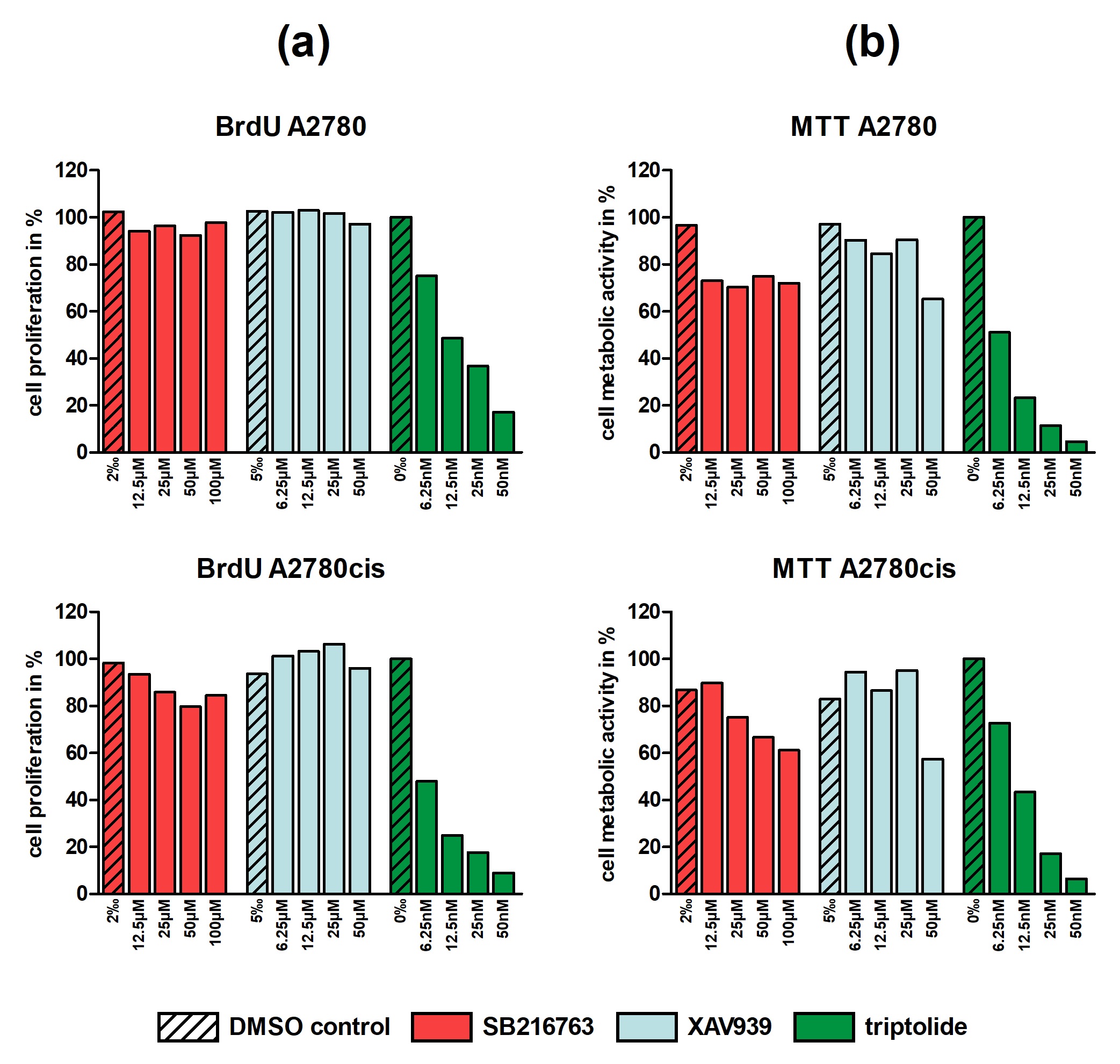

Supplement: Supplementary file 1 — Supplementary file1 (JPG 678 kb) [file 432_2020_3317_MOESM1_ESM.jpg]
